# A study protocol for a clustered randomised controlled trial to evaluate the effectiveness of a peer-led school-based walking intervention on adolescent girls’ physical activity: the Walking In ScHools (WISH) study

**DOI:** 10.1186/s12889-020-08600-0

**Published:** 2020-04-21

**Authors:** S. Maria O’Kane, Angela Carlin, Alison M. Gallagher, Ian M. Lahart, Russell Jago, Maria Faulkner, Marie H. Murphy

**Affiliations:** 1 Centre for Exercise Medicine, Physical Activity and Health, Sports and Exercise Sciences Research Institute, University of Ulster, Jordanstown Campus, Newtownabbey, BT37 0QB UK; 2 Nutrition Innovation Centre for Food and Health (NICHE), Biomedical Sciences Research Institute, University of Ulster, Coleraine Campus, Coleraine, BT52 1SA UK; 3 Faculty of Education, Health and Wellbeing, University of Wolverhampton, Walsall Campus, Gorway Road, Walsall, WS1 3BD UK; 4 Centre for Exercise, Nutrition & Health Sciences, School for Policy Studies, University of Bristol, Bristol, BS8 1TZ UK; 5 Department of Law and Humanities, Letterkenny Institute of Technology, Port Road, Letterkenny, Ireland

**Keywords:** Physical activity, Adolescent girls, Walking, Schools, Intervention

## Abstract

**Background:**

Adolescent girls in the UK and Ireland are failing to meet current physical activity guidelines. Physical activity behaviours track from childhood to adulthood and it is important that adolescent girls are provided with opportunities to be physically active. Walking has been a central focus for physical activity promotion in adults and may effectively increase physical activity levels among younger people. Following on from a pilot feasibility trial, the purpose of this cluster randomised controlled trial (c-RCT) is to evaluate the effectiveness of a novel, low-cost, peer-led school-based walking intervention delivered across the school year at increasing physical activity levels of adolescent girls.

**Methods:**

The Walking In ScHools (WISH) Study is a school-based c-RCT conducted with girls aged 12–14 years from eighteen schools across the Border Region of Ireland / Northern Ireland. Following baseline data collection, schools will be randomly allocated to intervention or control group. In intervention schools, female pupils aged 15–18 years will be invited to train as walk leaders and will lead younger pupils in 10–15 min walks before school, at break and lunch recess. All walks will take place in school grounds and pupils will be encouraged to participate in as many walks as possible each week. The intervention will be delivered for the whole school year (minimum 20–22 weeks). The primary outcome measure is accelerometer-measured total physical activity (counts per minute) (end of intervention). Secondary outcomes will include time spent in sedentary behaviour, light, moderate and vigorous intensity physical activity, anthropometry measures, social media usage and sleep. A mixed-methods process evaluation will also be undertaken.

**Discussion:**

The WISH Study will examine the effectiveness of a low-cost, school-based, peer-led walking intervention in increasing physical activity in adolescent girls when delivered across the school year. If the intervention increases physical activity, it would benefit adolescent girls in the defined target area with potential for wider adoption by schools across the UK and Ireland.

**Trial registration:**

ISRCTN; ISRCTN12847782; Registered 2nd July 2019.

## Background

Regular physical activity is associated with physiological and mental health benefits for adolescents including a reduced risk of obesity, improved fitness and cardiometabolic health, increased muscle and bone strength [1–3]. Despite this, globally, many children fail to meet current guidelines of 60 min of moderate-to-vigorous physical activity (MVPA) per day [4–6] and it is estimated that on the island of Ireland only 14% of post-primary school children meet the current recommendations [7]. Physical activity levels decline as children move into adolescence [8] and through to adulthood [7]. This decline is most pronounced among adolescent girls [9] where the average annual reduction in total physical activity from the age of 5 to 18 years is 4.6% [10]. Importantly, physical activity habits adopted during adolescence track into adulthood [11, 12] and may affect the likelihood of developing many chronic health conditions.

Schools are an excellent setting for physical activity promotion among adolescents [13] particularly as children spend 40% of their waking time at school [14], however, there is a lack of consensus on how best to promote physical activity within the school setting to ensure the maintenance of physical activity behaviours into late adolescence, and adulthood [13]. School-based Physical Education (PE) provides an opportunity for young people to participate in structured, regular physical activity [15] but research has shown that girls are offered significantly less PE time than boys [16]. In addition, extra-curricular physical activity within the school environment often reflects the content of the PE curriculum, i.e. team-based, structured sports [17] and girls are less likely to participate in such activities [18]. School recess may provide an opportunity to promote physical activity. Interventions during recess are feasible [19, 20] and female pupils tend to socialise with friends during this time but are less active than males so this period may provide a unique opportunity to increase physical activity levels in adolescent females [21–24].

Walking is the most natural form of physical activity [25] and has been recommended for the promotion of public health [26, 27]. Walking addresses many of the reported barriers to physical activity, such as lack of time, money or poor health [28] and is the most popular form of physical activity for adult women with over 50% walking for recreation each week [29]. A recent meta-analysis has demonstrated the beneficial effects of walking interventions on adult health [30], however less is understood about the potential of walking to promote physical activity in adolescents. The results of a recent systematic review outlined that walking interventions may provide an effective means for increasing walking in younger populations, at least in the short term but called for additional research into walking and physical activity in adolescents [31]. There is also evidence that walking has benefits for mental health, wellbeing and sleep quality [32, 33] but the evidence in the adolescent population is limited.

It is recognised that there are many barriers to participation in physical activity among adolescent girls including a perceived lack of time, peer pressure, negative school experiences, social media and lack of confidence [34–36]. Focus group discussions with adolescent girls have highlighted the characteristics of a school-based intervention acceptable to pupils to encourage participation among low active girls [37]. This work suggested activities that required no change of uniform and could be performed with friends, during the school day, were likely to be accepted and encourage participation [37]. It is recognised that enjoyment of physical activity is positively correlated with physical activity participation levels for adolescents [38] and research has shown that low-active girls enjoy non-competitive physical activity within the school setting and enjoy physical activity when participating with friends [39]. Research has shown that friends engage in similar levels of physical activity and physical activity interventions within the peer group may be effective as friendship can influences physical activity behaviour [40]. Furthermore, peer leadership is a promising strategy for influencing adolescent behaviour and increasing physical activity as peer leaders can motivate pupils to initiate and sustain behaviour change [41, 42].

Declining physical activity levels during adolescence may be attributable to various other factors including intrapersonal (e.g., self-efficacy, perceived competence, self-image), social (e.g., peer influence) and environmental factors (e.g., gender-relevant physical activity opportunities) [43]. It appears that during adolescence there is a lack of interest in the activities offered [44], girls are less likely to engage in organised sport [45] and there is a need to provide physical activity opportunities for those discouraged by the competitive selection process [46] which may increase physical activity in adolescent girls [44, 47]. Considering there is growing pressure on schools to improve academic standards and performance, there is a need for physical activity interventions to be delivered at school but outside of curriculum time [48] and in recent years, there has been growing interest in the promotion of physical activity during school recess [49]. As girls are less active during recess [22, 23, 49], this period may present an opportunity to promote physical activity. However, despite the potential of school recess to promote physical activity, there is a lack of intervention research in adolescent girls [19] and given the imperative to increase physical activity in adolescent girls, finding effective, sustainable, low-cost interventions is essential.

The aim of this cluster-randomised controlled trial is to evaluate the effectiveness of a novel, low-cost, peer-led school-based walking intervention, delivered across the school year, at increasing accelerometer-measured physical activity levels of adolescent girls. It is hypothesised that intervention pupils will increase daily physical activity and replace sedentary behaviour during the school day with walking.

## Methods

### Study design

The WISH study is a school-based clustered randomised controlled trial (c-RCT). The design of the project was informed by a feasibility pilot study [50] that used the Medical Research Council (MRC) [51] framework for complex interventions to develop a peer-led walking intervention. Specifically, following a systematic review of walking interventions in children and adolescents [31], focus groups were conducted to explore the attitudes of adolescents towards physical activity [37]. The findings of both the systematic review and focus groups were used to inform the design of the peer-led, school-based walking intervention [50] based on the socioecological framework and informed by Self-Determination Theory (SDT), which provides a framework for understanding and enhancing the motivational mediators of behaviour change [52–54].

**Fig. 1:**
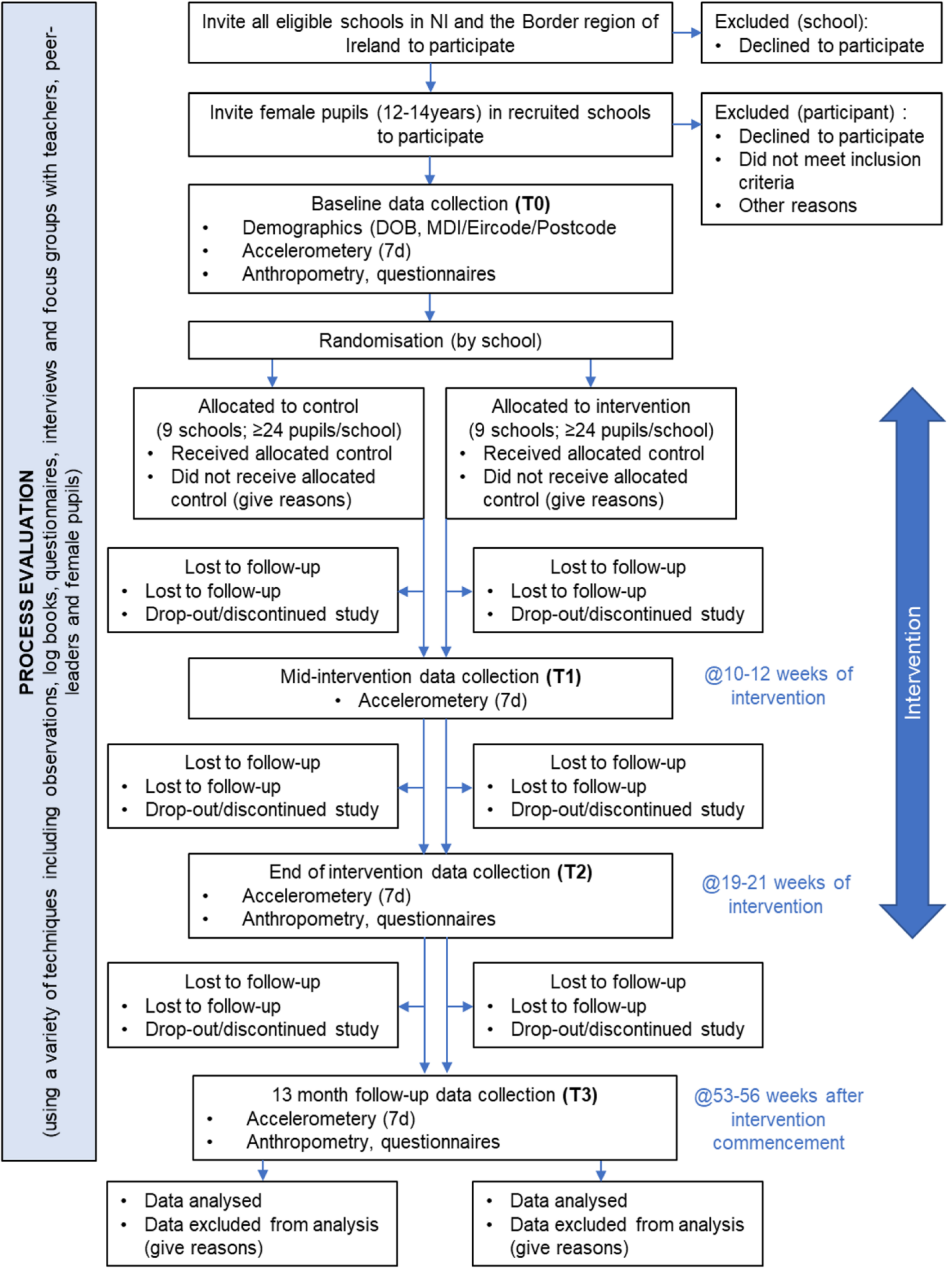
Study Flow Chart

Eighteen post-primary schools will be recruited across the Border Region of Ireland / Northern Ireland. Following completion of baseline data collection, schools will be randomised using a 1:1 allocation. Study outcomes will be assessed at four timepoints: baseline (T0), mid-intervention (T1), end of intervention (T2) and follow up (T3) as outlined in Fig. 1. A mixed-methods process evaluation will be undertaken at baseline and at the end of the intervention. Table 1 provides an overview of data collection and outcome measures, the details of which are provided below.

**Table 1:** WISH Study Data Collection and Outcome Measures

Outcome measure	Baseline (T0)	Mid-intervention (T1)	End of intervention (T2)	13-month follow-up (T3)
Demographic information (age, postcode/Eircode)	X			
Total physical activity (accelerometery)	X	X	X	X
Time spent in sedentary behaviour, and low, moderate and vigorous intensity physical activity (accelerometery)	X	X	X	X
Proportion of pupils meeting current physical activity recommendations (accelerometery)	X	X	X	X
BMI z-scores (height and weight)	X		X	X
Waist: hip ratio (waist and hip circumference)	X		X	X
Coping, resilience and cognitive reappraisal (Emotion Regulation Questionnaire for Children) [55]	X		X	X
Sleep quality, duration and efficiency (Pittsburgh Sleep Quality Index) [56]	X		X	X
Social media use, social integration and emotional connection to social media [57, 58]	X		X	X
Body weight and appearance satisfaction [59]	X		X	X
Self-efficacy for physical activity and walking [60]	X		X	X
Health-related quality of life (Kidscreen-10) [61]	X		X	X
Reasons for engaging in physical activity (BREQ-3) [62, 63]	X		X	X
Friendship nominations	X		X	
Process evaluation: focus groups with all pupils (control & intervention)	X			
Process evaluation: focus groups with intervention pupils			X	
Process evaluation: walk leaders - perception of physical activity and fitness [64], physical activity self-efficacy [65], physical activity enjoyment [66] and leadership skills [67]	X		X	
Process evaluation: interviews with walk leaders			X	
Process evaluation: interviews with teachers			X	

*Abbreviations*: *BMI* Body Mass Index

### Recruitment

#### School recruitment

The study setting will be post-primary schools in the Border Region of Ireland / Northern Ireland. Using school enrolment data [68, 69], schools that meet the following criteria will be invited to participate:

- Northern Ireland: Schools that have at least 80 girls across years 9–10 and located in Co. Derry/Londonderry
- Ireland: Schools in Co. Donegal with an enrolment of > 240 girls

Invitation letters accompanied with an expression of interest form will be sent to school principals. Where requested, a member of the research team will visit the school to provide a short overview of the trial for relevant staff. We aim to recruit 18 schools, of which 9 will be randomly allocated to intervention arm with the other 9 allocated to the control arm. Within each school, at least 24 children will be recruited (i.e., minimum of 432 children in total). Should it not be possible to recruit eighteen schools in the selected counties, schools that meet the inclusion criteria (Northern Ireland: at least 80 girls across years 9–10; Ireland: enrolment of > 240 girls) from other counties across the Border Region of Ireland / Northern Ireland will be invited to participate.

#### Participant recruitment

Following permission from the school principal, female pupils in Year 9/10 (Northern Ireland) and 1st/2nd year (Ireland) will be invited to take part in the study via a presentation from the study team which will inform pupils about the study, the randomisation process and intervention. In schools with < 70 female pupils in either Year 9/10 (Northern Ireland) or 1st/2nd year (Ireland), all girls in that cohort will be invited to participate. For the larger schools (> 70 pupils in either Year 9/10 (Northern Ireland) or 1st/2nd year (Ireland)), each school will be asked to provide a list of the form classes within Year 9/10 (Northern Ireland) or 1st/2nd year (Ireland) and for those schools with mixed ability form classes, classes will be randomly selected to attend the recruitment presentation. If pupils are grouped into form classes based on academic ability, we will randomly invite equal numbers of classes from each band (for example, top, middle and bottom) to attend the recruitment presentation. At a minimum, all potential pupils (and parents/guardians) will be provided with a copy of the participant information sheet. Parents/guardians will be asked to provide written consent. Written assent will be obtained from pupils. The study exclusion criteria are outlined below:

1. Male pupils are not eligible for inclusion in this study, as it seeks to assess the effectiveness of a walking intervention targeted at adolescent girls only.
2. Pupils who are unable to walk or for whom walking is contraindicated will not be eligible for inclusion as taking part in increased physical activity during the school day may not be suitable for this population.

In intervention schools, female pupils aged 15–18 years will be identified by staff and invited to train as walk leaders, lead the walks and participate in an interview at the end of the intervention. One staff contact per intervention school will be recruited for an interview at the end of the intervention and written informed consent will be obtained. Walk leaders will be provided with a participant information sheet and will provide written informed consent. Participants will be free to withdraw from the study at any point without giving reason. This study was approved by Ulster University Research Ethics Committee (Ref: REC/19/0020) on 21.06.2019.

### Sample size calculations

Sample size calculations are based on the WISH feasibility trial [50], which detected a mean difference of 11.41 min in total physical activity (light, moderate and vigorous intensity physical activity) measured using accelerometery between the intervention (2 schools consisting of a total of 79 children) and control (4 schools consisting of 84 children in total) groups. In order to detect a difference of 11.41 min total physical activity per day between groups, assuming a standard deviation (SD) (pooled intervention and control group SD) of 25 min in total physical activity, a power of 80%, a significance of 0.05, an average cluster size of 20 children, an intra-class correlation of 0.03 (calculated from pilot data [50]), and a coefficient of variation of 0.46, the sample size needed is 15 schools increasing to 16 schools (8 per study arm) to allow for 10% cluster attrition. To allow for 20% loss to follow up we will recruit at least 24 children per cluster (at least 384 children in total). Based on recruitment rates of 34% in our feasibility study [50], all schools that have at least 80 girls across years 9–10 (Northern Ireland) or total enrolment of > 240 girls (Ireland) will be eligible to participate in the study. From the Northern Ireland School Census [68] we estimate that 77 schools meet these criteria and based on 2016 school recruitment data for Ireland [69] 54 post-primary schools meet these criteria.

### Randomisation

After data collection, schools will be randomly allocated to control (*n* = 9) or intervention (*n* = 9). Randomisation will be stratified by country and random allocation will be performed by faculty staff who will be blind to school identity and independent of the study team.

### Intervention

Female pupils aged 15–18 years (peer role models) with few existing extra-curricular commitments within school will be identified by school staff and invited to train as walk leaders to lead younger pupils (aged 12–14 years) in 10–15-min walks before school and at break and lunch recess (maximum 15 walks per week (3 per day)). All walks will occur in school grounds and pupils will be encouraged to participate in many walks as possible each week. Walks will primarily take place outdoors but in adverse weather conditions, indoor routes will be used where possible. Each pupil will be issued with a reward card which will be stamped after each completed walk. These cards will allow pupils to self-monitor their attendance, and stamps can be exchanged for small rewards with low monetary value (<€1), for example, pens, pencils, rulers.

The training for walk leaders has been developed in consultation with the Physical Activity Co-Ordinator (Western Health and Social Care Trust) and is based on the *Walking For Health* Training (Public Health Agency & NI Health and Social Care Trusts). The training aims to provide an overview of the WISH Study, an understanding of health walks, the expectations of WISH Walk Leaders, knowledge of how to plan walks and keep walkers motivated. The training will also inform walk leaders of the support available for them in this role. Training will be delivered by the Trial Manager on school premises and will last approximately 3 h. The training session has been divided into five sections: 1) completion of consent forms and study questionnaires; 2) ice breakers; 3) presentation and group work to focus on ensuring walk leaders consider safety concerns of facilitating the walks and the importance of the walks being performed at a brisk pace, i.e. at a pace sufficient to elicit moderate intensity physical activity in pupils. Training will also include information on providing and encouraging social support amongst pupils to align with SDT; 4) Walk leaders will be provided with instructions on how to plan walk routes (agreed by teachers) and they will also draft the walking timetable and arrange a central meeting point; 5) Walk leaders will have the opportunity to practice leading a walk under the supervision of the Trial Manager. All walk leaders and will be provided with a training manual and asked to evaluate the training programme. Refresher training will be available throughout the intervention. Should a school arrange refresher training, this will last approximately 1 h and focus on the pace of the walk, encouraging social support and planning new/alternative walking routes. During the refresher training, walk leaders will have the opportunity to practice leading a walk and they will be provided with feedback on pace, route and social engagement. The walk leader training is theoretically underpinned by SDT and similar to the Bristol Girls Dance Project [70], the training facilitator will adopt an autonomy-supportive teaching style that strengthens walk leaders’ personal resources. Walk leaders will be encouraged to decide the format of the walks for example, the use of music or games and routes taken. They can also choose to introduce themed walks (for example, Santa walks at Christmas). Walk leaders will be able to self-select the walks that they would like to lead, and the timetable will be determined based on the preferences of the walk leaders. Within a supportive training environment, walk leaders will be provided with the skills and competence to run the walking programme within their school.

Two walk leaders will accompany each walk, one at the front and one at the back of the group. If possible, a third walk leader will be present in the middle of the group. Walk leaders will encourage the younger pupils, set the pace of the walk and ensure the safety of pupils. Walk leaders will be provided with wrist worn heart rate monitors (Mi Band 3, Xiaomi, China) to guide the pace of the walk. Walk leaders will be advised to check that their heart rate is > 135 bpm which indicates that they are walking at a brisk pace [71]. Walk leaders will also be trained to look out for other signs that they are walking at a brisk pace (breathing a little faster; feeling a little warmer; feel their heart beating a little faster; still able to hold a conversation) should they choose not to wear the heart rate monitor. The walk leaders will monitor the pace of the walk and regularly remind pupils that they should be walking at a brisk pace and although there will be variations in fitness and perceived effort, this is reflective of a walking group. For most pupils, walking at the same pace as the walk leaders will be of moderate intensity. Walks will take place in a one of the pre-planned routes around the school grounds. At the end of each walk, walk leaders will stamp each pupils reward card and note attendees to monitor compliance with the intervention. Pupils who are not enrolled in the study are permitted to join the walks and will be provided with a reward card which they can exchange for small value rewards. Risk assessments will be performed by a member of the research team and a member of school staff for each predetermined walking route.

Following baseline measurements, the intervention will be delivered for the whole school year. Allowing for holidays, exams, educational trips and other school events this may vary across schools however the intervention will be a minimum 20 and a maximum of 22 weeks. As part of the social support component of the intervention, walk leaders will be invited to be part of a separate closed social media (Facebook) group designed to include opportunities for social support in the form of sharing progress and useful information. In addition, social support and encouragement to continue will be provided to walk leaders via weekly updates from the research team which will include: strategies to address barriers to participation, recognising progress; links to websites and resources; and vignettes of support and advice from other walk leaders. These pages will only be accessible to those in the intervention and will be moderated by named members of the research team for data protection.

### Outcomes

#### Primary outcome

The primary outcome will be total physical activity (counts per minute) of pupils at the end of the intervention (T2) measured using the Actigraph GT3X accelerometer (Actigraph LLC, Florida) worn for 7 days. The device will be placed on an elastic waist band and pupils will be asked to wear the accelerometer at all times, removing it only for bathing, water-based activities such as swimming and when asleep. Pupils will be asked to wear the accelerometer on their right hip and to ensure that it is worn in the same position each day. Pupils will be asked to wear the accelerometer for seven consecutive days and will be included in the analysis if they have ≥2 valid weekdays of data (500 mins/day) [72]. During measurement periods, pupils will be asked to keep a log of when they wore the accelerometer and took it off to encourage compliance with the wear-time protocol as recommended by Trost et al, 2005 [73]. Minutes of total physical activity (light, moderate and vigorous) per day will be estimated using the Evenson cut-points [74]. A sampling epoch of 15 s will be employed during data collection. Periods of ≥60 min of zero counts will be categorised as ‘non-wear’ and removed.

#### Secondary outcomes

As outlined in Table 1, a number of secondary outcomes will also be assessed. It is also important to note, however, that there are no statistical power calculations for these assessments and as such all analyses will be presented with point estimates and 95% confidence intervals but without *p*-values. Accelerometer data will be used to calculate:

- Total physical activity (counts per minute) at mid-intervention (T1) and follow up (T3)
- Time spent in sedentary behaviour and light, moderate and vigorous intensity physical activity [74] at mid-intervention (T1), end of intervention (T2) and follow up (T3)
- Proportion of pupils meeting current physical activity recommendations [6] at mid-intervention (T1), end of intervention (T2) and follow up (T3)

The following secondary outcomes will be measured at baseline (T0), end of intervention (T2) and follow up (T3) to assess between group differences and changes over time:

- Height (cm) and weight (kg) will be measured to the nearest 0.1 cm and 0.1 kg, respectively, using a freestanding stadiometer (Leicester Height Measure), and digital scales (Seca 877) to calculate body Mass Index (BMI). BMI will be converted to an age-specific and gender-specific z-score [75, 76]
- Waist and hip circumference will be measured to the nearest 0.1 cm using an anatomical measuring tape and waist-to-hip ratio will be calculated.
- Coping, resilience and cognitive reappraisal [55]
- Sleep quality, duration and efficiency (The Pittsburgh Sleep Quality Index) [56]
- Social media use, social integration and emotional connection to social media [57, 58]
- Body weight and appearance satisfaction [59]

At baseline (T0) the following descriptive data will be collected:

- Date of birth
- Home postcode/Eircode to derive Index of Multiple Deprivation (IMD)

### Process evaluation

A mixed-methods process evaluation will be undertaken. At baseline (T0), end of the intervention (T2) and follow up (T3), pupils (aged 12-14 years) will be asked to complete a series of validated questionnaires to assess self-efficacy for physical activity and walking [60], health-related quality of life [61], reasons for engaging in physical activity [62, 63]. Friendship nominations will be assessed at baseline (T0) and at the end of the intervention (T2) to determine the effect of social networks on physical activity behaviour and intervention engagement. This will involve each pupil nominating school friends who are also participants in the current study. The number of friends to nominate will not be specified, although 10 lines will be provided on the form. Friendship networks will be constructed from these nominations [77].

At baseline (T0), focus groups will be conducted in all schools and pupils will be randomly invited to participate. The aim of these focus groups is to investigate pupils’ motivation for physical activity, barriers to physical activity and the influence of social media usage on their physical activity. At the end of the intervention (T2), high and low attendees from the intervention schools will be invited to take part in focus group discussions. These focus group sessions will enable pupils to share their experience of the WISH trial and assess any changes in behaviour pre- and post-intervention. The focus groups will also seek to identify factors that affected participation, motivation and enjoyment of the intervention.

The fidelity of each walk will be assessed through a self-report checklist completed by walk leaders. This will note attendees and assess walk duration and location (indoor/outdoor). To assess how elements of the environment may have affected delivery of the intervention interviews will be conducted with walk leaders. These interviews will be conducted at the end of the intervention (T2). Walk leaders will be asked to complete a brief set of questionnaires at baseline (T0) and end of the intervention (T2) to assess self-perception of physical activity and fitness [64], physical activity self-efficacy [65], physical activity enjoyment [66] and leadership skills [67] to profile the characteristics of those pupils who volunteer as walk leaders.

In addition, an in-depth interview will be held with one school contact (e.g. Head of Key Stage 3 / Head of Year or equivalent) per intervention school at the end of the intervention (T2) to identify key elements that might have affected implementation. Any adverse events will be recorded and reported to the Chair of the Trial Steering Committee and the Chair of the Ethics Committee.

### Data analysis

Data will be entered electronically on a secure file storage system and password protected. Data will be anonymised by assigning a unique identification number to each pupil.

#### Quantitative analysis

Outcome data will be reported in accordance with Consolidated Standards of Reporting Trials (CONSORT) guidelines [78]. The statistician performing the data analyses (IML) will be blinded to allocation throughout the study and statistical analysis will only be undertaken when all data has been collected (T3). Pupils will be included in the analysis regardless of compliance with the physical activity intervention. We will apply a multilevel statistical model using ML- win [79] to assess changes in total physical activity (mins per week) from baseline to post-intervention. Multilevel modelling will also be used for the change in total physical activity at other timepoints and secondary continuous outcomes adjusting the false discovery rate using the Benjamini-Hochberg Procedure [80]. Outcomes will be compared between the control and intervention groups using a 2-level multilevel model, with pupils nested within schools. The models will be adjusted for the child-level covariates (level 1): baseline total physical activity (minutes per week), age and BMI z-score; and the following school-level covariates (level 2): social economic status (postcode/Eircode). In the interests of parsimony, covariates will only be retained if their inclusion results in a significant improvement in fit statistics.

#### Qualitative analysis

Semi-structured interviews and focus groups will be audio-recorded, transcribed verbatim and anonymised before being coded. Thematic analysis techniques will be used to generate initial codes using NVivo [version 12] and these will be grouped to form themes for each cohort.

### Participant remuneration

All participating schools will be eligible to claim reimbursement for the use of their facilities and for teacher time (up to a maximum total of €400) and provided with a summary of project findings. At each time point, pupils will be provided with an incentive (earphones; water bottle; charger pack or sports top) when they have returned the accelerometer having worn it for a minimum of 2 days (€17 total cost). Pupils in the intervention group who take part in ten walks will be able to exchange their reward card for a small value incentive (<€1), for example, pen, pencil, ruler.

### Patient and public involvement

The views of low-active adolescents were instrumental in designing the WISH intervention [37, 50, 81]. In advance of the feasibility/pilot, 64 low-active adolescents were consulted on how best to encourage them to increase daily physical activity [37]. The findings informed the development of the school-based, peer-led walking intervention [50]. Following feasibility/pilot, 45 participants provided feedback on both intervention and research components [81]. On the basis of the pilot study, post-primary schools in Northern Ireland (*n* = 208) were surveyed to assess acceptability of a school-based walking intervention [81]. For the current study, Youth Advisory Group (YAG) meetings will be held on four occasions throughout the course of the study (Phase 1: June 2019; Phase 2: March 2020; Post-intervention: June 2021; End of Study: February 2022). Schools from both jurisdictions will be asked to invite pupils aged 12–14 years (participants) and 15–18 years (walk leaders) to the meetings. The YAG will be consulted on all aspects of the research, inform the delivery of the intervention and provide researchers with an understanding of what would encourage/discourage participation. The YAG will advise on resources for pupils and be actively engaged in the dissemination of the findings. In addition, the walk leader training has been developed in consultation with the Physical Activity Co-ordinator (Western Health and Social Care Trust).

### Dissemination of project findings

In order to disseminate the findings of the study we will hold two dissemination events, one in Northern Ireland and one in Ireland. These will be open to all stakeholders and will present the findings of the research and provide further information on how schools could implement the programme. In addition, all materials used in the intervention will be made available to control schools and to both Education Authorities for dissemination to all schools in the Border Region of Northern Ireland / Ireland. Peer-reviewed publications will allow the results to be disseminated to the scientific community and policy makers. All papers will be submitted for publication in open access journals.

## Discussion

To our knowledge, this is the first fully powered trial that will investigate the effectiveness of a peer-led brisk walking intervention in adolescent girls. The current study builds on a promising pilot trial [50] which confirmed the feasibility of our planned intervention. Patient and public engagement has been and will continue to be central to this study which enhances the acceptability of the intervention, ensures that research is relevant and increases the self-esteem of those staff involved in the project [82].

The WISH Study aims to address several gaps in the current scientific evidence for walking interventions in adolescents. Firstly, until now there have been a limited number of studies which investigate the longer-term effectiveness of physical activity interventions, particularly in children and adolescents [31]. The current study includes longer-term follow up and will measure physical activity at 13 months’ post-intervention to address the paucity of evidence on the longer-term effectiveness of physical activity interventions in children and adolescents. Secondly, the WISH Study aims to provide adolescent girls with extra opportunities to be active at break and lunchtime and given the limited number of structured walking recess-based interventions [31], this study will address this specific gap in the current evidence base. In terms of study methodology, it is estimated that there are more than twelve methods available for measuring physical activity [83] and although there is no universally agreed “gold standard” method [84–86] the use of accelerometers enables us to objectively measure physical activity and provide information on the intensity, duration and frequency of physical activity [85, 87].

In accordance with MRC guidelines [88], the research team will undertake an in-depth mixed method process evaluation and the perspectives of multiple stakeholders (i.e. pupils, walk leaders and teachers) will be sought. This will enable a robust evaluation of how the intervention was implemented and an accurate interpretation of either positive and/or negative outcomes [89, 90].

To conclude, if the WISH intervention increases physical activity, there is potential for the programme to be widely implemented by schools resulting in a sustainable, long-term, positive impact on adolescent population health.

## Data Availability

Not applicable at this point.

## Abbreviations

CONSORT: Consolidated Standards of Reporting Trials
c-RCT: Clustered Randomised Controlled Trial
BMI: Body Mass Index
IMD: Index of Multiple Deprivation
MRC: Medical Research Council
MVPA: Moderate-to-vigorous physical activity
NICHE: Nutrition Innovation Centre for Food and Health
PE: Physical Education
SD: Standard Deviation
SDT: Self-Determination Theory
WISH: Walking In ScHools
YAG: Youth Advisory Group
